# Towards Intelligent Safety: A Systematic Review on Assault Detection and Technologies

**DOI:** 10.3390/s25133985

**Published:** 2025-06-26

**Authors:** Vikash Shankar Shyam Sundar Bhuvaneswari, Mohanraj Thangamuthu

**Affiliations:** Department of Mechanical Engineering, Amrita School of Engineering, Amrita Vishwa Vidyapeetham, Coimbatore 641112, India; cb.en.u4are21045@cb.students.amrita.edu

**Keywords:** assault prevention, wearable devices, mobile healthcare, remote monitoring, GIS, security framework, AI/ML

## Abstract

This review of literature discusses the use of emerging technologies in the prevention of assault, specifically Artificial Intelligence (AI), the Internet of Things (IoT), and wearable technologies. In preventing assaults, GIS-based mobile apps, wearable safety devices, and personal security solutions have been designed to improve personal security, especially for women and the vulnerable. The paper also analyzes interfacing networks, such as edge computing, cloud databases, and security frameworks required for emergency response solutions. In addition, we introduced a framework that brings these technologies together to deliver an effective response system. This review seeks to identify gaps currently present, ascertain major challenges, and suggest potential directions for enhanced personal security with the use of technology.

## 1. Introduction

The rising cases of physical violence, particularly against women and the downtrodden, point towards an imperative need to deploy sophisticated technologies for personal safety. Several systems based on IoT and smartphone monitoring have been framed to meet the exigent concern by providing immediate alerting solutions and quick responses in case of emergencies [1]. Wearable technology, smartphones, and sensor systems have been very effective in providing personal protection through tracking location, sending alerts in case of emergencies, monitoring health continuously, and quick connectivity with emergency numbers. Some mobile apps target the prevention of violence by using GPS tracking, fingerprint identification, and real-time sharing of locations. For example, apps, such as “Anti-Molestation” and “Women Safety Mobile App”, employ location alerts, voice recording, and emergency helpline functionality to facilitate victim protection [2,3]. Furthermore, IoT-enabled devices with built-in accelerometers and smart sensors can automatically recognize assault-like physical activities and initiate emergency responses without human intervention [4]. It minimizes response time considerably and maximizes the chances of rescuing the victim.

In addition, crowdsourcing platforms are useful violence prevention tools. The platforms enable users to anonymously report incidents of assault, which helps in the identification of violence hotspots and enhances law enforcement response [5]. Blockchain technology has also been suggested to protect victim information and maintain confidentiality, reducing the chances of information leakage [1]. Although the possibility of these technologies is vast, issues, like false alarms, excessive development costs, and poor accessibility in rural communities, remain [2]. Overcoming these is important for maximizing the impact by providing affordable, simple-to-use, and widely accessible safety systems.

This paper conducts an extensive review of literature across the intersection of AI, IoT, healthcare management, and prevention of assault with a focus on technological developments and their implications. It critically reviews current AI and IoT-based solutions, determines essential trends, issues, and loopholes in previous research, and proposes a conceptual framework for AI and IoT fusion to improve healthcare systems and individual safety features considering policy and ethics. The research is conducted to answer the following questions:

RQ1. What types of technological frameworks and systems are currently being used to enhance personal safety and emergency response?

RQ2. How can technology improve women’s safety through better crime detection and response?

RQ3. Can mobile apps, like the Women Safety (WoS) App, improve emergency response and safety for women in distress situations?

By responding to these questions, this paper seeks to provide useful knowledge for researchers, policymakers, and practitioners striving towards more effective and secure healthcare systems.

## 2. Methodology

This study employs a systematic literature review (SLR) methodology to consolidate and critically evaluate recent technological advancements in the field of assault prevention and emergency response. The focus is on applications involving Artificial Intelligence (AI), the Internet of Things (IoT), Geographic Information Systems (GIS), and wearable technologies. The SLR approach ensures structured identification, selection, and synthesis of relevant literature, thereby promoting transparency, replicability, and objectivity in drawing insights. To ensure comprehensive coverage of the domain, literature was sourced from several high-quality academic databases, including IEEE Xplore, PubMed, Scopus, ScienceDirect, MDPI, SpringerLink, and Google Scholar. These databases were selected based on their relevance to interdisciplinary research in technology and safety systems. Searching multiple databases minimized the risk of missing key studies and helped include both technical and applied research.

A structured keyword search strategy was implemented using Boolean operators. Example search strings included terms, such as “Assault Prevention” OR “Violence Detection” OR “Women Safety”, “IoT” OR “Wearable Devices” OR “GIS” OR “Mobile Applications”, “Emergency Response” AND “AI” AND “Healthcare”, IoT OR device with sos for assault prevention and IoT device with sos for women, men, child assault prevention. The search was restricted to studies published between January 2015 and December 2024 to reflect contemporary developments in the field. The inclusion criteria encompassed peer-reviewed journal articles and conference papers published in English that focused on technological interventions for assault prevention, safety monitoring, and emergency response. Eligible studies had to demonstrate real-world or simulated applications of AI, IoT, wearable sensors, GIS-based mapping, or mobile healthcare tools. Exclusion criteria ruled out duplicate records, non-peer-reviewed literature, theoretical-only frameworks lacking validation, and inaccessible full-text articles.

The initial search retrieved a large body of literature. After duplicate removal, titles and abstracts were screened for relevance. Studies meeting the criteria were reviewed in full text, and only those providing significant insights into technological safety solutions were retained. A data extraction template was developed to systematically collect key information from each selected study. Extracted data included the type of technology, target population, regional scope, system architecture, performance metrics (like accuracy, latency, and power consumption), and reported challenges. The reviewed studies were then grouped into thematic categories: personal safety and wearable devices, IoT-based attack detection systems, GIS-enabled safety frameworks, and mobile applications with AI integration. Where available, comparative metrics were analyzed to assess the effectiveness, efficiency, and scalability of the proposed systems.

## 3. Assault Prevention

### 3.1. Personal Safety

Five core security uses in domestic violence prevention were identified by Prenzler et al. home security, shelter security, GPS tracking, duress alarms, and multi-systems. Home security upgrades, such as the safe@home program, were found to effectively decrease fear and physical attacks. Duress alarms, such as Bsafe and AWARE, enhanced victim safety, albeit with limited access. The Bradford Staying Put Project and Staying Home Leaving Violence illustrated that the incorporation of security with legal and advocacy services increased protection. Nevertheless, most interventions did not undergo thorough scientific assessment, constraining absolute conclusions on long-term efficacy [6]. Sathyasri et al. talk about how the system improves women’s safety by incorporating GPS, GSM, and IoT for real-time location tracking and emergency notification. When activated, it sends an SMS with GPS coordinates to registered numbers and police and updates the location on a webpage. A neurostimulator provides electric shocks to discourage attackers, and a buzzer warns people around [7].

**Figure 1:**
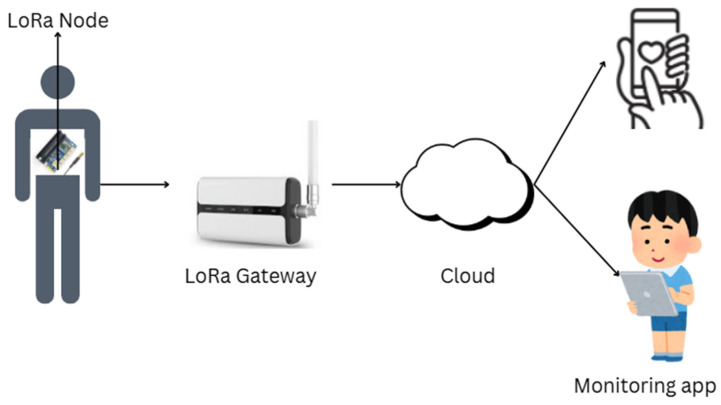
The network diagram of the proposed wearable sensor network.

Wu et al. present the efficiency of the WE-Safe project, which exploits an IoT self-powered sensor network built using LoRa technology to undertake real-time monitoring of environmental parameters, and the network diagram is shown in Figure 1. It harvests solar energy for perpetual functioning on ultra-low power consumption. It continuously monitors risky environmental situations safely through the exploitation of wearable sensor nodes featuring micro-power management. It transmits real-time alerts through a mobile application and, in doing so, provides the user with enhanced security levels in unsafe areas. Test results confirm good network coverage, outdoor transmission up to 520 m, and good reliability [8].

**Figure 2:**
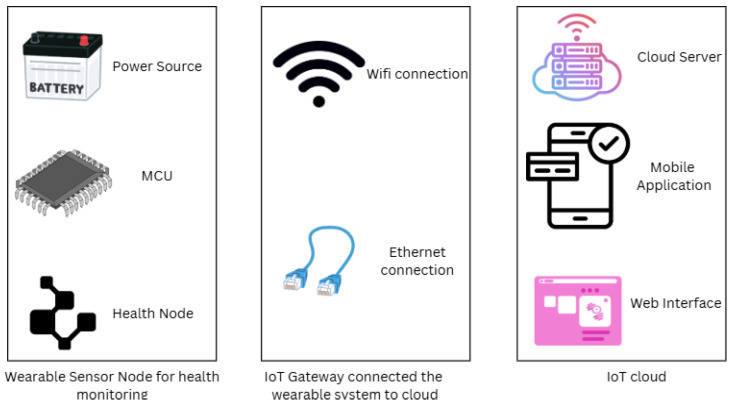
System architecture of the wearable sensor network.

Rodríguez et al. suggest an integrated ICT solution for Intimate Partner Violence (IPV) management, coupled with IoT and Machine Learning (ML) for managing data, communication, and survivor protection. The system provides ongoing surveillance, which enhances symmetry in monitoring and intervention. The system emphasizes the privacy and security of survivor information and grants approved personnel access to multiple channels of communication. The architecture of the wearable sensor network is shown in Figure 2. Machine learning algorithms interpret data for risk analysis, which improves real-time protection and support. The research underscores that IoT and ML complement each other to enhance IPV management [9].

Sadhu et al. give an extensive overview of IoT security threats and solutions, categorizing attacks and vulnerabilities in IoT systems and addressing conventional and innovative security mechanisms, such as cryptographic approaches and Physical Unclonable Function (PUF)-based techniques. It presents challenges in IoT security and suggests recommendations for future work, encompassing the taxonomy of IoT security and privacy problems, centralized and decentralized security solutions, and countermeasures against various attacks. The research underscores that IoT applications continue to be top targets for attackers, and vulnerabilities cut across device-level, network, and data transport attacks [10].

Salami et al. address IoT processing limitations through offloading techniques, suggesting the SOS-FCI scheme for secure mutual authentication. It is resistant to active and passive attacks effectively and offers secure session key generation for communication. Performance analysis with the NS3 tool indicates its improved security features over other comparable schemes, though packet loss and delay are greater with more devices connected. It comprises six various operational stages that enhance the security of IoT offloading using robust authentication, encryption methods, and resistance to cyber-attacks [11]. Bhatia et al. identify the threats of cyber-surveillance via IoT devices and suggest an anomaly detection strategy to prevent misuse. It introduces the MFEW anomaly detection model for IoT systems that classify normal and abnormal usage in real time. It uses a hybrid feature selection approach combining filter and wrapper methods to optimize relevant features. Ensemble learning via bagging enhances accuracy and robustness. The system integrates both proactive and reactive strategies to counter IoT-based abuse, addressing the challenges of high-dimensional, unstructured data. The MFEW Bagging approach identifies normal and anomalous behavior through ensemble feature selection and attains 99.8% accuracy for the NSL-KDD dataset considering only 10 features. Attackers use IoT for invasion of privacy, and real-time mitigation strategies are therefore important [12].

Madhav et al. write about the smart hospital, which utilizes a LAN with star topology for high-speed, dependable communication between IoT devices. Some of the principal systems are fire and smoke detectors, RFID-based ward doors, motion and temperature sensors, and computer-controlled lighting and alarms. Hospital operations and data exchange are supported by a web server, FTP, and email services. The network is scalable, and flexible, and employs DHCP for dynamic assignment of IPs. Safety, efficiency, and high-quality healthcare delivery are the focus of the design [13].

RQ1. What types of technological frameworks and systems are currently being used to enhance personal safety and emergency response?

Zahra et al. respond to IoT security and privacy issues through the introduction of GLSF2IoT, a lightweight and scalable approach that identifies misbehavior through Fuzzy Logic and Fog-based architecture. It identifies blackhole, selective forwarding, collusion, and DDoS attacks efficiently while adding negligible memory overhead. The system functions in uncertain and heterogeneous IoT environments with a zero-trust model applied to all nodes and ongoing misbehavior monitoring. Performance results indicate better accuracy compared to current benchmarks. The research stresses that security cannot be an afterthought in IoT, sounding the alarm on digital annihilation with no strong safeguards [14].

Eranpurwala et al. refer to the deteriorating situation of women’s safety in India with crimes against women being commonly reported. The essay points to the widespread existence of gender-based violence across the world, equating its severity with large-scale health crises. The research elaborates on the implications of violence against women for society and the increased demand for justice by way of street protests. For improved safety for women during the crisis, an application is under development that focuses on the immediate requirement for technology-driven interventions. The app requires user registration with personal details and includes an emergency SOS button to alert contacts. During emergencies, it provides continuous location updates and captures audio clips and images. User ratings on localities help generate safer routes, and AI with machine learning supports intelligent decision-making. The study highlights the imperative for action-based approaches towards ensuring an end to violence against women and strengthening their security within both public and private spheres [15].

Desai et al. investigate the overall dichotomy of technology regarding women’s safety since it delivers some advantages but also runs risks. While mobile apps and digital means may intensify security, they nevertheless create vulnerabilities attaching to the very potentials existing nowadays due in large part to an altogether different cybercriminal element. Security awareness, weltering in the arena of social media exposure, more capitalistically produced than physically concentrated, among its other aspects, sadly occurred because of insufficient cybersecurity awareness and has proved to be a hotbed of cyber threats against women. Mobile applications and smartwatches offer GPS and GSM integration for real-time tracking and emergency alerts. They store location-wise incident histories to inform users about unsafe areas. Apps, like HARASS map, use interactive maps for reporting harassment, and alerts with location details are sent to registered contacts during emergencies. The research identifies certain gaps in legal and social measures currently addressing online crimes as well as the viability of existing safety applications. There lies the crux of the necessity for creating awareness, improving legal frameworks, and initiating preemptive strategies toward fortifying women against any digital and physical threat [16].

Mohsin et al. propose an IoT-based intelligent emergency response system combining vehicle, home, and health monitoring to augment public safety. The system employs IoT-based smart technology for real-time emergency response, integrating vehicle, home, and health monitoring. It uses sensors, like GPS, accelerometer, temperature, flame, humidity, gas, and health sensors, for heart rate and oxygen levels. Data are processed via NodeRed and Laravel and stored in a MySQL database, and the system provides automatic emergency notifications through a user-friendly interface. The system has a dramatic response time improvement of 3 milliseconds and over 99% accuracy in emergency detection using sophisticated sensors and cloud processing. It has real-time data acquisition, predictive analytics for risk management, and ease of use with a friendly interface [17].

Shelby et al. engage in questions around the involvement of technology in preventing gendered violence, critique the punitive ethos, analyze the technoscientific inequalities based on gender, and work within the frameworks of abolitionist justice, focusing on how those realities are sometimes racialized. The analysis includes key identified missed opportunities; it could more unabashedly and engage with how technology might somehow help shape abolitionist and transformative justice agendas, through a lens of analysis related to race and class status. It critiques in what sense some of these interventions, specifically anti-violence technologies, focus on categorical gender identity to focus on populations of college-aged people that are lost and left aside because of social input and the experiences of persons in intersectional/lower-class/race-based situations [18].

Bellini et al. analyze abusive partners’ (APs’) understandings of technology abuse in Abuse Partner Intervention Programs (APIPs). The study finds that APs tend to excuse their behaviors while denying personal responsibility, posing a challenge for intervention. With the help of discursive psychology and critical discourse analysis, the research investigates the ways APs describe their experience and how societal factors influence the narratives. It is difficult for practitioners to confirm APs’ accounts, thus creating distrust and disengagement. The research identifies reporting gaps and a dearth of practitioner training in tech abuse, with implications for the need for specialized interventions. The results indicate ethical principles and better technology design as a way to strengthen abuse prevention initiatives [19].

Vimal et al. discuss Traffic Analysis and Fault Detection (TA/FD) measures to protect weak points of networks and recommend an enhanced encryption approach for IoT devices with compliance under the GDPR. The work exploits the application of Software-Defined Networking (SDN) in upgrading routing algorithms and power saving, adopting Secure and Safe Internet of Things (SerIoT) approaches to advanced IoT encryption and access management. Some gaps noted include weak points of mobile network controllers, the unpredictability of clustered networks, and a lack of hazard detection methodologies. Reliable routing protocols and trust assessments are vital for secure IoT communication. The SDN-based SernCPN architecture improves energy efficiency and control in Cognitive Protocol Networks. Stochastic Neural Networks support decentralized decision-making, while RNNs and ISM data aid in machine learning. Security is reinforced through assurance assessments, reliability testing, traffic forecasting, and Fused Goal Functions to optimize QoS and protection [20].

Simpson et al. analyze how GPS tracking incorporated into a system aims for the betterment of child well-being while simultaneously highlighting privacy, autonomy, and family structure. Since the technology offers a safety net for parents, it may unintentionally intimidate independence and resilience in a child. The research offers a critique against the playing out of the elements involved as simple and linear to just an ultimate good or bad taken by the technocracy and their respective dance of moral concerns on the other side concerning surveillance. Following this, whether tracking ensures safety or reinforces societal fears shall be looked into regarding how children negotiate freedom. Finally, the paper proposes an urgent need for the integration of a paradigm shift in understanding the constructive and destructive interplay between protection and autonomy within a technology-based society [21].

Othmani et al. survey artificial intelligence and machine learning approaches for the diagnosis and assessment of Post-Traumatic Stress Disorder (PTSD), with a focus on video and EEG sensor data. It addresses the limitations of traditional PTSD diagnosis methods, such as the imprecision of standard questionnaires, and underscores the potential of AI-driven methods in improving diagnostic accuracy, particularly in telemedicine contexts. EEG recordings and video-based methodologies are explored as key modalities. EEG data are utilized for classification tasks, although their scarcity and lower sensitivity compared to video data pose challenges, especially in preserving patient identity. Eight EEG-based studies are identified, highlighting the current scope and limitations in the field. Video analysis leverages deep-learning techniques to process temporal frames of 25 ms duration, where deep neural networks extract high-level features from facial images, particularly action units, which are crucial for PTSD assessment. Additionally, video restoration techniques are employed to mitigate degradation effects and improve data quality [22].

Khan et al. represent a significant advancement in multimodal emotion recognition (MER), particularly when deployed on consumer devices. By integrating audio and visual modalities through joint feature representations and multi-scale transformer architectures, the JMMT model effectively captures both inter and intra-modal relationships. This synergy enhances recognition accuracy, especially for subtle or underrepresented emotions, such as “fear” and “disgust.” The model has been validated on benchmark datasets, like IEMOCAP and MELD, where it consistently outperformed state-of-the-art methods in terms of both accuracy and F1 score. Its hierarchical recursive fusion mechanism and attention-based temporal modeling further strengthen its robustness in real-world scenarios. Practical implications of JMMT include improved user experiences in customer service, telehealth, and social media sentiment analysis, where emotion recognition is pivotal. Moreover, the model is optimized for deployment on edge devices using techniques, such as pruning, quantization, and ONNX standardization, enabling real-time, energy-efficient inference without sacrificing performance. Despite its strengths, the model faces challenges, like misclassification of similar emotions (e.g., frustration vs. anger) and sensitivity to data imbalance and quality. Future directions emphasize enhancing the model’s adaptability for real-time applications, expanding to additional modalities, and refining performance for imbalanced data distributions [23].

Lin et al. discuss the integration of artificial intelligence (AI) into biomedicine, which has revolutionized the analysis of omics data, enhancing precision in genomics, proteomics, transcriptomics, and metabolomics. In computational genomics, AI models have improved gene prediction, splice site recognition, and enhancer identification by leveraging large-scale sequence data. Proteomics benefits from AI in predicting protein structures, most notably through AlphaFold, and in identifying protein–protein interactions and subcellular localization. In transcriptomics, AI facilitates the detection of differentially expressed genes and cell-type-specific expression through feature extraction and clustering techniques. Metabolomics applies AI to decipher metabolic profiles from mass spectrometry data, aiding drug discovery and disease diagnosis. However, challenges, such as data imbalance, ethical concerns, and the need for high-quality, long-term datasets persist. Despite these limitations, AI continues to offer transformative potential in biomedical research by unlocking insights from complex biological data [24].

Chattopadhyay et al. explore the development of a machine learning-based Clinical Decision Support System (CDSS) for diagnosing and grading typhoid fever, a disease with rising antibiotic resistance and diagnostic challenges. Ten virtual junior clinicians (VJCs), each powered by different machine learning classifiers (MLCs), were trained using a synthetic dataset of 198 cases curated by physicians. Symptoms were weighted and modeled using Gaussian distributions, enabling the VJCs to learn from a clinical rule base. Random Forest (VJC9) emerged as the most accurate model, outperforming both other algorithms and human clinicians. The study highlights the potential of ML in enhancing early, accurate typhoid diagnosis, especially in resource-limited settings. Challenges, such as explainability, synthetic data reliance, and limited real-world validation, remain. However, the proposed CDSS supports novice clinicians and aligns with broader efforts to integrate AI into clinical workflows [25].

Ogunjobi et al. explore that chronic diseases, such as diabetes, cancer, and cardiovascular disorders, remain major global health challenges, demanding advanced strategies for prevention, diagnosis, and treatment. This review explores the role of bioinformatics and machine learning (ML) in chronic disease research, with a focus on genomics, transcriptomics, proteomics, and metabolomics. Genomic data from GWAS and NGS helps identify risk variants, biomarkers, and therapeutic targets, enabling precision medicine. Transcriptomic profiling and multi-omics integration reveal dysregulated genes and pathways involved in disease pathogenesis. Proteomics uncovers post-translational modifications and protein interaction networks, crucial for biomarker discovery. ML applications enhance early diagnosis, prognosis, and personalized treatment strategies. Real-world case studies and translational research demonstrate the clinical impact of these tools. Despite progress, challenges in data quality, patient privacy, and equitable access remain areas for future research [26].

El Saddik et al. provides an in-depth survey of generative AI (GenAI) techniques used to create multimodal content images, videos, audio, and 3D assets within the Metaverse. It reviews foundational models, including GANs, VAEs, diffusion models, and normalizing flows, detailing their roles in generating interactive avatars, environments, and digital twins. The findings highlight GenAI’s potential to enhance immersion, creativity, and personalization in virtual environments. It includes the successful application of GenAI to dynamic avatar generation, procedural scene creation, and adaptive audio synthesis. Limitations include high computational costs, lack of standardization, real-time processing challenges, data availability issues, and ethical concerns related to ownership, bias, and privacy. The paper emphasizes the need for scalable, interoperable solutions to fully realize GenAI’s potential in the evolving Metaverse [27].

### 3.2. Attack Prevention

Nagaraj et al. propose a machine learning-based assault mitigation system that tracks victims’ locations and alerts authorities using GPS, GSM, and fingerprint modules to enhance women’s safety. The system employs GPS and GSM modules for real-time location tracking and an Arduino board as the core processor. Real-time monitoring enables high-accuracy violence detection, and the system predicts assault locations using historical data while sending emergency alerts to police and relatives. A fingerprint sensor ensures user authentication before activation. It sends SOS messages to emergency contacts and uses machine learning to predict potential assault locations. Designed for low-cost implementation, the system supports real-time monitoring and violence detection. The system is presented as low-cost and effective for emergency purposes, but it also offers possibilities for public protection services. Current women’s safety systems are known to have a gap, including high expense, failure in function, and an absence of event information, such as location and time, which bring into question their efficacy both at home and outdoors. These flaws point toward a need for lower-cost, simpler-to-use safety devices [28].

Polamurı et al. cover the growing threat of physical attacks on systems from the accelerated growth of connected devices and the Internet of Battlefield Things (IoBT) by 2025. Current anti-tamper designs are based on pre-defined responses, which do not perform well against stealth attacks. To mitigate these shortcomings, the authors suggest a machine learning-based intelligent anti-tamper system that discriminates between normal and abnormal behavior, minimizing false alarms and maximizing security. The system features an autonomous anti-tamper design using machine learning to detect suspicious behaviors. It employs sensors to monitor physical parameters and uses algorithms, like Random Forest, SVM, K-NN, and Isolation Forest, for behavior classification. An analytical system labels data as normal or attack, and a tiered reaction and recovery mechanism ensures an appropriate response to tampering. The system incorporates a tiered reaction mechanism and recovery strategy to enhance operational reliability. Because of the projected increase in hostile capabilities, the research points to the importance of adaptive anti-tamper solutions to protect critical infrastructure [29].

Kumar et al. introduce a new string coordinating strategy algorithm to improve Network-based Intrusion Detection Systems (NIDS) for better cybersecurity. It elaborates on two NIDS modes promiscuous and network system and utilizes several sensors to observe network behavior. The new system improves intrusion prevention by neutralizing misuse instances in real time. The research is cited to emphasize the cost of cyberattacks, which is estimated at up to USD 1 trillion by 2020. It points out existing gaps, such as insufficient detection of zero-day attacks and safeguarding mobile agents. The report highlights the importance of scalable AI-based analytical modules to improve security for IoT devices as well as cloud networks [30].

Nagaraju et al. propose a hybrid optimization scheme and deep learning paradigm to boost cybersecurity in IoT environments. It provides solutions to threats, such as illegal downloading and malware attacks, by combining Grey Wolf Optimization and Whale Optimization algorithms to enhance intrusion detection efficiency. The proposed approach improves feature selection, diminishes the dimensionality of data, and maximizes classification accuracy compared to current methods. Primary difficulties encompass dataset shortages in training IDS, difficulty in processing deep learning models, and conventional detection limitations by multi-type IoT networks. The system integrates a hybrid optimization mechanism combining Grey Wolf and Whale Optimization for enhanced detection accuracy. A Deep Convolutional Neural Network (DCNN) is used for malware analysis, featuring convolution, pooling, and fully connected layers. The Adam optimizer and Softmax Cross-Entropy loss function optimize training, while data preprocessing improves signal quality and reduces noise. It was emphasized that complex preprocessing schemes are required in the research area in addition to robust cybersecurity infrastructure in IoT [31].

Sharma et al. propose a Zero-Trust mechanism as a countermeasure to cyberattacks. It examines privacy, confidentiality, and trust as key safety challenges from the perspective of motivation and means of attackers: intelligence agencies and private firms. This describes vulnerability issues in IoT security: software bugs, bad testing, and open physical access points that can be exploited. It calls for further research on challenges and solutions to the cybercrime concern to fill the knowledge gap and enhance security for IoT [32]. Goyal et al. investigate IoT security by using Raspberry Pi as a honeypot to study attack patterns and user intention. Three honeypots, namely Cowrie, Dionaea, and Glastopf, were set up on Raspberry Pi 4B to observe and defend against vulnerable IoT protocols. The system uses Raspberry Pi 4B running Raspbian OS as a honeypot to monitor IoT attacks. Cowrie for SSH/Telnet, Dionaea for malware capture, and Glastopf for HTTP-based threats, like port scans. Logs are analyzed using Natural Language Processing to detect and understand attack patterns. The results prove the usefulness of honeypots in detection and threat analysis, aiding in the increased security measures [33].

Khan et al. introduce THASSA, a secure hardware-based trusted architecture for IoT systems that off-loads crypto operations to a special device to alleviate host processor loads. It leverages a COTS TPM for secure storage of keys and crypto execution to provide strong message exchange and dynamic rolling of keys. Experimental findings confirm the efficacy of THASSA in secure data transfer, with crypto offloading enhancing performance but trust extension adding some delay. THASSA architecture features a trusted device alongside IoT devices, using lightweight encryption (AES, Tiny Encryption Algorithm) to reduce overhead. Cryptographic tasks are offloaded to the trusted device, which ensures message integrity, detects tampering, and validates digital signatures. Custom message formats and encrypted secure channels protect communication, while confidential Trust IDs prevent unauthorized access. The architecture is scalable, allowing several trusted hardware devices to create secure IoT clusters while ensuring communication integrity [34].

Vardhan et al. propose a computer vision and CNN-based anomaly detection system to improve campus-wide college safety. It identifies smoking, acts of vandalism, and physical violence through live surveillance. CNNs are more accurate and precise than conventional SVMs for intricate image classification. Graph-based contextual analysis refines anomaly prediction and prevention. Ethical considerations regarding surveillance as well as data privacy are noted [35].

### 3.3. Geographic Information System

Shenoy et al. suggest an integrated system for women’s safety based on combining crime analysis, prevention, and emergency response. It includes a mobile application (SpotHer), a quick alert wearable device, and a WebGIS system for analyzing crime hotspots. Sophisticated GIS methods detect high-risk zones, using socioeconomic data and crime pattern histories for enhanced crime mapping. Real-time SOS alerts, artificial intelligence-powered risk assessment, and user location tracking were tested with success to facilitate timely emergency response. The framework improves crime surveillance, prevention, and cooperation among law enforcers, enhancing public safety through cutting-edge technology, data insights, and an engaged community [36].

RQ2. How can technology improve women’s safety through better crime detection and response?

Suresh et al. suggest a holistic framework for women’s safety by incorporating crime analysis, prevention, and emergency response. It utilizes mobile app data and wearable devices along with GIS technology to detect crime hotspots and trends. The system focuses on community participation to strengthen safety features and fill the loopholes in current crime prevention mechanisms. The main contribution is the incorporation of different crime-related services for an integrated response. The research also points out weaknesses in existing systems, including connectivity and inadequate community participation, emphasizing the importance of a more integrated and efficient strategy [36].

Paradkar presents intelligent security systems that enhance women’s safety through mobile apps, wearable devices, and AI-driven solutions. The existing systems are classified into mobile applications with emergency features, wearable microcontroller-based devices, and AI-powered security solutions utilizing facial recognition and movement analysis. The proposed model is a complete safety solution with SOS alerts, GPS tracking, GSM communication, spy camera detection, and intrusion alerts [37]. Kommey et al. propose a crime-reporting mobile application to improve real-time crime reporting and public safety in Ghana. The app allows users to report crimes from afar, saving time that would otherwise be spent visiting police stations. Accurate location tracking, enhancing emergency response times is provided by GPS. The system also monitors reported incidents and enables communication between users and law enforcement agencies. The system features user registration and allows users to file, view, and report crimes in real time. Police stations can register their details and locations, while users can attach evidence to support their reports. The application is built with clearly defined functional and non-functional requirements to ensure usability and effectiveness. While the application supports criminal investigation, it does not yet have a report generation system for data analysis [38].

Pavate et al. introduce a machine learning-driven safety route system to alleviate women’s safety issues in Delhi. It finds 15 to be an optimal number of clusters, and crime rates are graphically depicted using color-coded symbols. The research paper explores the use of the k-means clustering algorithm to predict safe routes for women in crime-prone areas, aiming to enhance accuracy and reduce access time. Trained on 1481 records and tested on 634, the system achieved 79.7% accuracy with a 70 ms response time. K-means groups similar data points to distinguish safe from unsafe routes effectively. Its unsupervised nature and compatibility with various data types make it suitable for handling large datasets. The research points out that the safety of women is still a major issue in Delhi, calling for data-based solutions to provide more security and confidence [39]. Kamilaris et al. discuss IoT applications that utilize geospatial analysis techniques, with a focus on the use of location data in environmental knowledge. It classifies IoT applications into six geospatial analytical approaches and finds 55 applicable projects utilizing these approaches. The research also points out limitations, like errors in interpolation and kriging due to gaps in data, spatial data infrastructures’ lack of standardization, and incompatibility between mapping platforms. Moreover, limited implementation of interoperability standards for IoT networks impedes data exchange. The study highlights the necessity of enhanced geospatial methods and standardization towards maximizing the performance of location-aware IoT applications [40].

Gargiulo et al. analyze the impact of perceived safety on the utilization of green spaces (GE) by women for physical activity, and it is pointing towards gender disparities in accessing GE. A pilot study using qualitative GIS (qGIS) established a map of safety through intensive interviews, and 90% of the observable space was found to be perceived as dangerous. Environmental characteristics, such as lighting, vegetation cover, and land use, were found to influence perceptions of safety. The results indicate that better lighting and urban planning can facilitate women’s access to GE. The research also highlights methodological shortcomings in safety evaluations, calling for additional research on extensive GE. Managing these variables can foster gender-friendly policies and motivate women’s outdoor physical activity [41].

Jeon et al. suggest a Big Data and IoT solution to minimize sexual offenses in Korea by processing crime data and warning people in risk zones. It discusses the transformation of crime prevention from CPTED to smart systems and emphasizes the contribution of ICT developments towards safety improvement. The system employs text mining and machine learning techniques to detect risky places. Some of the principal challenges involved IoT security breaches, data inconsistency, and infrastructural instability. It stresses that effective implementation would be dependent upon policy and technical advancement. It offers findings for a more intelligent, data-guided crime reduction program [42]. Yi et al. investigate the effect of GPS-based walkability and greenspace exposure on physical activity during pregnancy and postpartum in Hispanic women. Results indicate that higher exposure to parks and walkable neighborhoods is associated with higher moderate-to-vigorous physical activity. The research points out the limitations of conventional residential-based exposure measures and emphasizes the importance of taking into account non-residential settings. Outcomes favor urban planning interventions to improve greenspace access, especially in low-SES neighborhoods. Although results suggest positive correlations, the limited sample size and cross-sectional nature of the study constrain causal inferences [43].

### 3.4. Wearable Device for Safety

Bhargavi et al. speak of how the COVID-19 pandemic propelled the innovation of IoT-based solutions to aid public health and safety. It encompasses social distance tracking through BLE and ESP32, hand sanitizer dispensers that automatically dispense, and wearable health monitors. BLE provided low-power proximity notifications, and ESP32 sensed neighboring devices to aid in enforcing distancing. Contactless sanitizer systems and real-time health monitoring minimized human contact and enhanced hygiene. IoT devices also facilitated remote health monitoring and quarantine enforcement. IoT technologies were effective during the pandemic and are worth their weight in gold post-COVID [44].

Wu et al. discuss the IoT-based wearable sensor system improves safety in outdoor workplaces through environmental condition monitoring and physiological health monitoring. It combines a wearable body area network (WBAN) with a low-power wide area network (LPWAN) for stable communication. A local server in real-time processes sensor signals, presents data, and initiates emergency notifications. The system successfully monitors parameters, including heart rate, temperature, UV exposure, and CO2 levels. Experimental outcomes verify secure data transmission and network coverage that provides constant monitoring in industrial settings [45]. Márquez-Sánchez et al. discuss the value of intelligent PPE for the improvement of employee safety and minimizing accidents via smart wearable technology. The intelligent system combines a helmet, a bracelet, and a belt featuring sensors for online monitoring and detecting anomalies. New advanced artificial intelligence methods enhance the forecasting capability and make proactive protective actions and threat avoidance possible. Application of the 5G network provides enhanced speed for transmitting data, promoting timely and smooth communication. The latest wearable technology enables round-the-clock monitoring of an individual, remarkably enhancing work safety in high-risk sectors, like construction, mining, and metallurgy [46].

Additionally, Márquez-Sánchez et al. present an intelligent multisensor bracelet to detect anomalies and monitor user activities in real time. The system comprises a smart multisensory bracelet, helmets, and belts to monitor health parameters in real time, featuring sensors, like a Type-K thermocouple and an optical heart rate sensor. It uses AIoT with machine learning models GMM and LSTM for accurate activity classification and anomaly detection. Alarms are triggered through sound and vibration upon detecting risks, with data sent to a server for continuous monitoring. The platform, refined for ergonomics and response time, has been validated across diverse work environments. It advances safety in transport, mobility, and logistics through the detection of traffic anomalies and reducing accidents. The system implements adaptive learning that allows independent classification of data so that accurate and reliable monitoring takes place. The latest machine learning architectures, such as Long Short-Term Memory (LSTM) and Gaussian Mixture Model (GMM), enhance the precision of anomaly detection. The study attained 92% accuracy in the classification of non-daily activities, showing efficient and high-quality data collection. The ergonomic design of the bracelet enables customization for different uses, enhancing workplace and personal safety considerably [47].

Hyysalo et al. discuss the Smart Mask that bridges IoT, AI, and sensors to achieve real-time health monitoring for improving COVID-19 protection as well as respiratory safety in multiple settings. It updates a centralized model based on local training results to increase individual performance without sharing data, providing privacy and efficiency. The considerations are latency, connectivity, battery life, and security for continuous operation with reliability. The Smart Mask is a basis for proactive and reactive healthcare applications, such as pandemic readiness. Subsequent research is warranted on adaptive machine learning models, pricing models, material technology, and comfort for the user to maximize adoption and long-term use [48]. Humaira et al. present wearable safety gadgets, BOHNNI and BADHON, to improve the security of women in Bangladesh amidst increasing incidents of harassment and violence. BOHNNI, a locket-shaped device, includes GPS, GSM, and Bluetooth modules to send alerts with location data. BADHON, a bracelet, features a video camera and a non-lethal shock mechanism (up to 10 mA) to deter attackers. Both are lightweight, made from polymer plastic, and were tested across 14 body placements to ensure quick activation in emergencies. The gadgets use GPS and GSM for tracking and emergency signals in real time, triggered by voice recognition for ease of use. The important features are a shocking module to disable aggressors and a response time of 1.95 s, with 91.67% accuracy for reliability [49].

Anish Choudhary et al. introduce a smart wearable device that will provide an augmentation of women’s security based on GSM and GPS technology. The device will allow the user to send a distress signal immediately by pressing a button, thus notifying family members or police in no time about the present location. Integrated with the ATmega-328 microcontroller, the device would effectively function; it also anticipates being miniaturized in the form of jewelry or within a mobile device, a piece of wearable technology usable in daily life. The system enables rapid emergency response through a button press that triggers SMS alerts to contacts and authorities, along with a buzzer to attract nearby help. A built-in GPS module shares the user’s real-time location, aiding quick rescue. It features a neurostimulator to temporarily incapacitate an attacker and uses GSM to send continuous alerts until resolved. An LCD provides real-time feedback on system status and alert activity. The work is focused on the urgent need to provide such systems for women’s safety society-wide, particularly in India, in which women would need more security due to social taboos. The system would have the capability of empowering women through abuse detection in near-real time with rapid deployment in emergencies [50].

Anandhi et al. researched enhancing women’s safety and security, an IoT-based wearable device that offers enhanced security for women in various settings. The hybrid network incorporates GPS, heart rate, vibration, tilt, and shock sensors for detection of distress conditions and automatic SMS to police and relatives. An SOS button is also provided for manual communication of the distress signals. In comparison to the current safety solutions, it is light, cheap, and friendly while evading the drawbacks that come together with heavy and expensive alternatives. Real-time IoT connectivity further enhances monitoring with the capability of intervening fast and ensuring the secure movement of women [51]. Priya et al. propose a rapid-response safety system for the protection of women in unfortunate situations. The system is made up of an Arduino UNO microcontroller, including GPS for location tracking, GSM for SMS notifications, a push button to switch it on, an LCD, and a buzzer for all alarm sounds. When the woman is threatened, she presses the button, which sends an alert with real-time location updates to defined emergency contacts. The integration with IoT and features for self-defense, like an electric shock module to scare off attackers, are also explored by the authors. The authors stress the importance of these kinds of technological solutions to advance the safety of women, especially in a public transport system [52].

Magidwar et al. introduce a wearable safety jacket that aims to promote women’s safety by integrating self-defense and alert features. The jacket has a panic button to alert authorities and relatives, GPS and GSM modules for tracking, and a non-lethal electric shock system to scare off assailants. The system also takes pictures of the attacker as evidence powered by an ATmega2560 microcontroller and a 12 V rechargeable battery. It features GPS for location tracking, GSM for sending emergency alerts, and includes a screaming alarm, electric shock mechanism, and audio/video recording for self-defense and evidence collection. The system activates via a switch and communicates with emergency contacts through a smartphone and Glassfish server. The jacket allows real-time monitoring and violence detection, which sends alerts with location information to registered contacts in case of harassment. However, the research reveals reliability gaps in GPS and GSM, particularly in cloudy weather and extreme indoor conditions. The study indicates the necessity of enhanced protective technologies, highlighting women’s safety as a top social issue [53].

Wa et al. focus on violence and crime as threats to residence safety and property, highlighting family ties in minimizing fear perception. The study proposes Famiband, a wearable gadget against street robbery, and highlights the influence of interaction modalities on functionality and usability. Psychological safety emerges as an important factor in city life, and the study emphasizes that community trust plays a vital role in alleviating fears from crimes. Yet, it highlights areas of research, including the requirement for outdoor-based real-world testing, deeper examination of design aspects, such as shape and materials, and an enhanced understanding of how wearable technology might improve safety perception over mobile phones. The research ultimately seeks to enhance urban security and user trust through novel personal safety technology [54].

**Figure 3:**
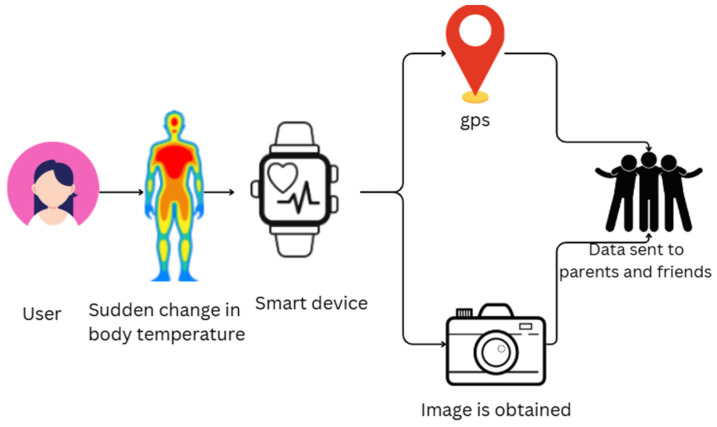
Architecture of the IoT wristband.

Phadatre et al. propose an IoT-based emergency alert gadget with real-time sharing of location and health monitoring capabilities, such as tracking temperature and pulse rate. It increases citizen safety through the use of community volunteers for rescue missions. The gadget sends alerts to emergency contacts when a double press on a button is performed, sending updates of location every five seconds. Yet, the drawbacks are the size of the bracelet, minimal testing in small communities impacting volunteer availability, and reliance on the user’s phone for sending notifications. Also, possible volunteer response time delays are mentioned. The research emphasizes the role of societal responsibility in providing citizen safety through innovative IoT technologies [55]. Ebenezer et al. present an IoT-enabled wristband that aims to make women safer by continuously keeping track of their vital signs, like pulse, temperature, and vibrations. The system incorporates sensors and uses the Blynk app for real-time notifications, which alert family members in the event of an emergency. There is also an ESP32 camera that helps in gathering situational information for effective incident handling. The architecture is shown in Figure 3, which helps to transmit the data to the family. The overall objective is to utilize IoT technology to deliver an active security solution for women in society [56].

Arvinda et al. introduces an IoT-enabled wearable gadget, intending to improve women’s safety through real-time monitoring, tracking, and emergency response capabilities. The system combines Raspberry Pi, sensors, and GPS to allow constant tracking of location and instant alertness in case of emergencies. It also has SOS signals, enabling parents to easily trace children and giving a feeling of security in daily life. Wireless technology makes remote access possible, and the device integrates several safety features, including defense mechanisms. The research identifies the critical need for security solutions based on IoT to empower women and ensure personal safety in all settings [57]. Zuha Maksood et al. offers a personal safety device prototype based on GSM technology to resolve escalating security issues. The device incorporates an Arduino Uno, SIM900 GSM modem, and an LDR sensor for the functionality to send distress SMS messages. Affordability and accessibility inform the design as locally sourced materials are used. Testing proved that the device would operate successfully to facilitate emergency communication. Yet, the research finds a lack of post-incident victim support and calls for more efficient crime prevention measures beyond situational awareness and emergency numbers [58].

Gupta et al. demonstrate an intelligent system utilizing cutting-edge technology, such as machine learning, GSM, and GPS, to improve the safety of women during emergency scenarios. The notable features are the sharing of live locations, one-touch emergency messaging, and evidence capturing through audio and video. The research points out loopholes in current safety systems, including the lack of real-time identification of perpetrators, no integration with emergency services except police (hospitals and fire brigades), and safe route discovery for women traveling alone. To fill these gaps, a “Smart Band” device is suggested, which will offer real-time support and enhance overall security arrangements for women [59]. Telagam et al. introduces an intelligent safety device for women that leverages IoT and virtual instrumentation technology to monitor the location and provide emergency alerts. The device has real-time GPS location tracking, voice recording for investigations, and group messaging features, which run independently of a mobile phone. The system, which can be triggered by voice commands, provides location updates at two-minute intervals and brings together multiple safety functionalities in a single device. They have been designed to augment women’s safety, overcoming the imperatives of past safety apps by enabling women to move around freely without fear while providing instant response during emergencies [60].

Islam et al. propose an IoT system powered by machine learning for the real-time prevention of sexual harassment, based on wearable force-sensitive resistors (FSRs) integrated into clothes. The sensors’ data are sent to the cloud and processed by a pre-trained AdaBoost classifier, and the system maintains 99.3% accuracy. The system alerts authorities in real time through a mobile app upon recognizing harassment. Gaps identified are possible false alarms, calibration issues, power limitations, and internet reliance [61]. Nagrare et al. focus primarily on security vulnerabilities within the pairing and service phases using the STRIDE threat modeling framework. A few key threats are those of DoS attacks, spoofing, and tampering, with correctness in BLE implementation emphasized. A quantitative framework is discussed for improving security, addressing some pertinent issues of weak encryption and application-level weaknesses in UUID structures. The intention is to ensure that ongoing security attestation and cryptographically reinforced authentication mechanisms are critically important to keep vulnerabilities at bay [62].

Revathi et al. introduced a complete women’s safety system that incorporates various technologies for improving security, using an Arduino Uno microcontroller. The system consists of an SOS button, temperature sensor, pulse oximeter, vibration motor, and GPS tracking for instant emergency response. A Nerve Stimulator is used to produce vibrations, gaining attention in times of distress. Machine learning algorithms are used to support anomaly detection and threat prediction, enhancing preventive measures. The study highlights the imperative for tailored safety measures for women in unsafe settings [63].

### 3.5. Mobile Application for Safety

Adithya et al. present a mobile application, Ally to enhance individual security by SOS messaging in real time and tracing of locations. Developed on Flutter with Firebase hosting, Ally allows users the facility to dispatch panic messages using fingerprints or voice, within 30 s. Ally uses Twilio messaging and Mapbox for the last seen location upon poor network situations. Some of the main features involve a survival manual, grabbing media in times of distress, and crowdsourced assistance from local users. The app fills gaps in existing safety solutions by offering a rapid, private, and scalable system of emergency response [64].

Farooq et al. find that there is a rising trend in the interest in women’s safety gadgets for IoT, with the greatest development in research activity post-2019. Enhanced automatic triggering of alarm systems for enhanced efficiency is required, as indicated by its focus. Certain sensors, such as pulse rate and pressure sensors, are utilized extensively, while machine learning programs are used to detect threats. Whereas 35% of the studies were higher than average in terms of quality assessment, 24% were lower than average. Validation was performed in 59% of the studies to ensure their reliability. Categorization of the studies recorded an interrater reliability of 0.92, which confirmed the robustness of the findings [65].

Arya et al. propose a solution for women’s safety as an IoT and machine learning-based solution to identify threats in real time. Physiological stress measures, like wearable EEG sensors and eye blink detection, are employed to identify indicators of fear or anxiety. Machine learning algorithms process labeled data to detect likely threats and issue immediate alarms. Ethical considerations, such as reducing algorithmic bias and ensuring privacy in data, are emphasized. The research calls for this system to be combined with existing available safety infrastructure to expedite emergency response. The accessibility and affordability of the technology are also prioritized to make the technology inclusive for different socioeconomic classes [66].

Daga et al. present Suraksha, an intelligent security system that aims to improve women’s safety through the use of smartwatch technology. With the integration of sensors, like accelerometers, gyroscopes, heart rate sensors, and GPS, the system can automatically identify distress situations and send out emergency alerts. Location information is sent to registered contacts, and a first-aid guide offers instant help. The study calls for automatic activation in safety systems to overcome current limitations. Interdisciplinary collaboration is advocated to enhance applicability and viability. Future studies will seek to extend the capabilities of the system, investigating more safety situations and technological innovations [67]. Rodríguez et al. summarize 73 studies on the use of technology to combat violence against children and women (VAC and VAW). Online detection describes 50.7% of studies, which are on detecting abusive content using machine learning. Safety solutions, such as IoT-based wearable and mobile apps, make up 26% of studies. Offline detection (11%) and education tools (12.3%) are also used for preventing violence. Different sources of data, including legal records and social media, contribute to the formulation of AI-based solutions. The research highlights the role of computer science technologies in fighting VAW and VAC [68].

Karmakar et al. introduce FemmeBand, an intelligent security band made for emergency alarm applications, featuring EMG and heart rate sensing to gather data. The application uses a wireless body area network (WBAN), sending out SOS messages accompanied by GPS position to mobile platforms when emergencies strike. Based on experiments carried out under standard conditions, the system shows increased efficiency compared to previous models. Its prospects involve the implementation of advanced wrist sensors, applying NODEMCU ESP8266 for greater diversity and adopting AI for decision-making and execution [69].

Zargar et al. came up with a women’s safety scheme that is technology-based, suggesting a prototype system that utilizes GPS, Beacon technology, and IoT to ensure real-time monitoring of safety. The system comprises sensors that monitor unusual circumstances, track important parameters, and initiate warnings to the family and police along with the location. While maintaining safety and reliability, the paper charts vital loopholes in current systems, including the absence of constant network connectivity and difficulties victims incur while communicating in situations of panic. The cost-efficient framework improves women’s security through an effective infrastructure for emergency intervention and real-time location in dangerous situations [70]. Rajkumar et al. discuss IoT-based women’s safety devices that employ scream detection and video recording to improve security in risky situations. It highlights the need for automatic alert systems that provide location information during emergencies while calling for AI integration to enhance danger prediction and ease of use. Some of the key issues in current systems are sensor sensitivity leading to false alarms, dependence on network connectivity that restricts use in remote locations, and battery life and cost issues that impact mass adoption. The paper emphasizes the need for dependable, real-time monitoring and automatic detection systems to inform authorities promptly, highlighting technology’s critical role in women’s safety solutions [71].

RQ3. Can mobile apps, like the Women Safety (WoS) App, improve emergency response and safety for women in distress situations?

Yadav et al. present WoSApp, a mobile app to increase women’s safety by providing clandestine emergency calls via phone shaking. It tackles police response limitations, including finding crime locations and victims’ lack of ability to contact authorities secretly. WoSApp sends GPS location and emergency contact information automatically to the police, supported by Mangalore Police through a special helpline. The system is intended to enhance the efficiency of emergency response, eliminating confusion during times of distress. Pilot testing among female students garnered positive reviews, pointing to its potential as an effective safety aid [72]. Vinarao et al. researched an application that provides safety for women using GPS tracking, SMS notification, and emergency calling to police stations. The application creates awareness of crime prevention and assists the police in handling emergencies. Performance testing verifies its reliability, efficiency, and usability, enhancing perceptions of user safety. The research identifies policy gaps in discussions of women’s security and the social tendency to offload safety responsibility on women. The findings underscore the necessity of integrated policies and technological intervention to deal with issues of safety efficiently [73].

McCarthy et al. research deeply analyzes personal safety apps and their effect on the safety of public transport and privacy issues. All respondents were willing to download such an app, but females were reluctant due to privacy reasons. Younger users responded negatively to location tracking, and police monitoring increased perceived safety greatly. Gaps in efficiently addressing demographic issues and ethical issues in testing safety apps are emphasized through this research. Research indicates an emerging demand for customized safety technologies that harmonize security, privacy, and ease of use for public transport [74].

Table 1 provides a categorized overview of recent advancements in personal safety technologies, focusing on wearable devices, mobile applications, and supporting systems. It includes the WE-Safe sensor network for real-time environmental monitoring, cybersecurity measures for IoT attack prevention using deep learning and bio-inspired optimization, GIS-based frameworks for proactive crime prevention, hybrid wearable systems for health and safety monitoring, and AI-driven mobile applications aimed at detecting and preventing violence against women and children. Each category outlines innovations, advantages, and critical challenges, such as power dependency, privacy concerns, integration complexity, and system scalability, offering a comprehensive perspective on current capabilities and limitations in the domain of safety technology.

Table 2 presents a detailed comparison of comprehensively summarized key metrics, such as performance, power consumption, and cost. This table provides a clear comparison of these factors, offering valuable insight into the trade-offs and efficiencies of the evaluated systems. By presenting these parameters side-by-side, Table 2 serves as an essential reference for understanding the overall effectiveness and practicality of the solutions discussed.

### 3.6. SoS Framework

**Figure 4:**
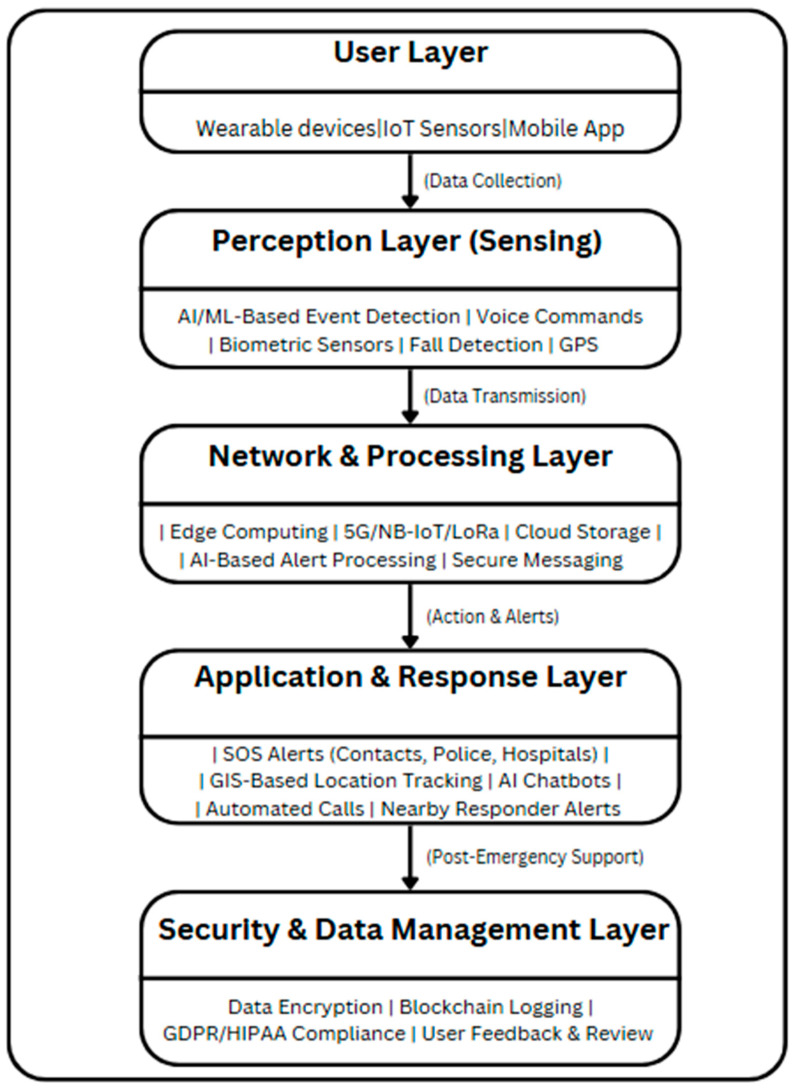
SoS Framework.

The System of Systems (SoS) Framework is an AI-driven emergency response framework leveraging wearable sensors, mobile applications, and IoT-based monitoring systems. The framework is delivered at multiple-layer levels in real time to detect, process, and react to emergencies while ensuring security, privacy, and reliability. The SoS Framework enables manual and AI-based distress (or alert) activation, biometric monitoring, data transfer or transmission in a secure way, AI-driven alerts involving decision-making automation, and automated emergency response systems. The SoS Framework also uses edge computing, encryption, and blockchain for computing efficiency, data accuracy, and compliance with privacy demands governed by regulations. The framework is shown in Figure 4.

User Layer: This layer bridges various devices and applications that the user would interact with: wearable sensors, mobile applications, or IoT-based monitoring systems. The types of devices will allow activating distress signals in two unique ways: manual SOS activation (button press, shake detection) and automatic detection (fall detection, biometric anomalies, or AI-based voice recognition).

Perception Layer (Sensing): The layer collects data from different sensors and systems for health emergencies or personal safety acts detecting threats. AI/ML algorithms analyze biometric information, motion patterns, and voice recordings to check for emergency events. For instance, in the health monitoring case, the biosensors detect abnormal vital signs using some abnormal characteristics, such as a high heart rate or sudden falls. In the case of assault prevention, through AI, a voice recognition system detects specific distress phrases, hence triggering an alarm. Likewise, during this stage, GPS location data will also be captured to support responders.

Network and Processing Layer: Once the emergency is detected, the data gets processed and sent through a secure and efficient network infrastructure. Low-latency processing is ensured by the use of edge computing, which processes distress signals locally before sending them to the cloud. Information transmission in remote or resource-constrained environments is uninterrupted through communication technologies, like 5G, NB-IoT, or LoRaWAN. Additional AI-driven alert processing eliminates any false alarms, ensuring only genuine emergencies are escalated.

Application and Response Layer: This is the action center of the SOS framework in which emergency notifications are sent automatically to specified contacts, police agencies, or health professionals. GIS-based tracking systems enable real-time monitoring of the location of the user such that responders can establish their precise location. AI-based chatbots or virtual assistants may communicate with the user to gauge the seriousness of the situation and assist them until help arrives. The system may also serve to alert nearby volunteers or community responders able and willing to offer immediate assistance.

Security and Data Management Layer: For data integrity and privacy, the system applies end-to-end encryption, logging on blockchain, and adherence to legal data protection laws, like GDPR or HIPAA. Incident reports are retained securely for potential future analysis, predictive analysis, and AI model improvement. User feedback for that specific emergency response can better aid continuous precision and efficiency enhancement of the system.

### 3.7. Proposed Solution/Methodology

1. Wearable and IoT Sensors—Smartwatches, rings, or biosensors for vital sign monitoring, fall detection, and distress signal activation.
2. AI-Based Distress Detection—Machine learning models analyze heart rate, motion, voice patterns, and environmental data to detect emergencies.
3. Edge and Cloud Computing—Edge devices process distress signals locally for low-latency responses, while cloud servers store historical data for analysis.
4. GIS and Location Tracking—GPS-enabled tracking ensures real-time responder navigation to the victim’s location.
5. Automated Emergency Alerts—SOS messages are sent to emergency contacts, law enforcement, and nearby community responders.
6. Secure Communication and Data Encryption—Ensures privacy protection and compliance with GDPR (General Data Protection Regulation)/HIPAA Health Insurance Portability and Accountability standards for healthcare data security.

**Figure 5:**
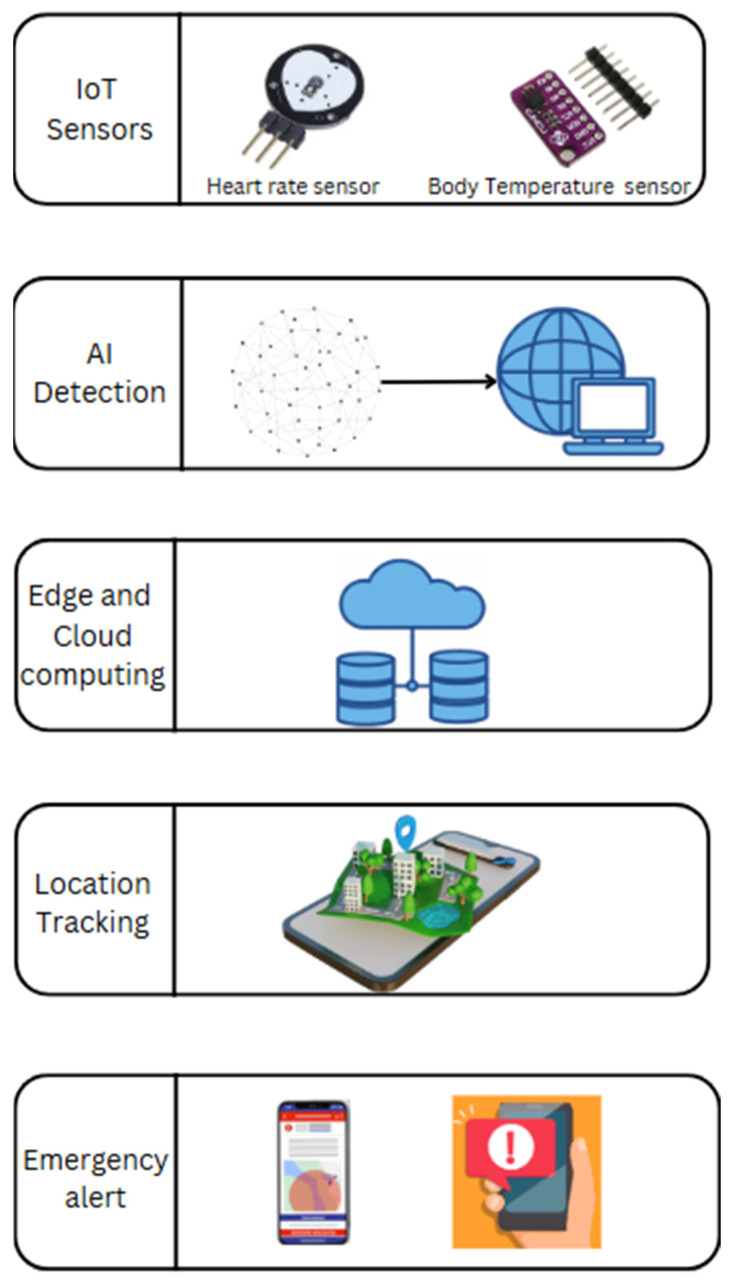

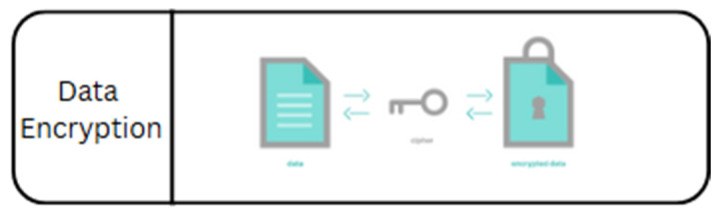
Key components of the SoS Framework for AI-driven emergency response.

Figure 5 depicts the fundamental elements of the System of Systems (SoS) Framework, combining wearable and IoT sensors for real-time safety and health monitoring, AI-driven distress detection to recognize emergencies, and edge and cloud computing to process data efficiently with low latency. GIS-based tracking allows for precise responder navigation, facilitating rapid response. Automated emergency notifications alert emergency contacts, law enforcement, and community responders. Moreover, data encryption and secure communication protect privacy as it maintains consistency with GDPR as well as HIPAA guidelines for safeguarding confidential healthcare data along with secure incident reporting.

## 4. Implementation

The SOS solution is applicable across industries, such as healthcare, personal protection, public areas, and law enforcement. In hospitals and old-age homes, it facilitates real-time monitoring of patients, identifying medical distress situations, such as falls or unusual vitals. In public transport, schools, and offices, wearable SOS devices and mobile apps increase security, which enables users to send emergency signals in situations of risk. Smart cities can couple the system with CCTV, GIS tracking, and emergency services for quick response. Law enforcement agencies can use the framework for community-based safety networks that alert nearby responders. The anticipated benefits are quicker emergency response, AI-based decision-making, and fewer false alarms. The application of edge computing and cloud integration provides low-latency processing and data security. The architecture is cost-effective and scalable and can thus be used in various environments. The use of encrypted communication and blockchain-based logging provides privacy and support for GDPR/HIPAA regulations. As a solution that can improve safety in real time, the SOS architecture provides an effective and intelligent healthcare and assault prevention solution with the potential to save lives.

### 4.1. Challenges

The SOS framework is vulnerable to false positives and detection accuracy because AI models are prone to giving false alarms that need constant adjustment to enhance dependability. Delays in network connectivity in the field can affect emergency response times, but edge–cloud hybrid processing can alleviate such an issue through real-time processing locally. Another key issue is data security and privacy, requiring end-to-end encryption and GDPR/HIPAA compliance to safeguard user data. Another challenge is user adoption and awareness, as individuals must be trained in the advantages of the system through training and awareness programs. Lastly, scalability and integration are also important considerations, as the framework needs to accommodate multiple devices, applications, and emergency response networks, and hence a modular and flexible architecture for easy expansion.

### 4.2. Future Recommendations

For advancing the SoS Framework further, future enhancement will include adopting context-aware AI models that review diverse data streams, like biometrics, motion, and environmental cues, for enhanced accuracy of emergency identification as well as diminished false alarm frequency. Making use of 6G technology, satellite networks, and ultra-wideband technologies will provide efficient network connectivity in remote or catastrophe-exposed places. Blockchain-based logging can also be used to improve data security, guard against tampering, and enable verifiable incident reports for legal and medical purposes. Furthermore, AI-driven, personalized emergency response plans customized based on individual health conditions, location, and risk factors can optimize response efficiency. Extending the framework to smart city infrastructure, like CCTV, traffic management systems, and public safety networks, can offer real-time situational awareness for prompt emergency responses. Implementation of sophisticated edge AI models will enable real-time detection of distress right on devices with reduced dependency on cloud computing and enable immediate alerting in low-connectivity conditions. Next-generation systems will need to support multi-modal emergency interfaces, such as wearable haptics, AR/VR interfaces, and voice assistants based on AI, to develop more natural and user-friendly mechanisms to trigger emergency response and provide aid to the users effectively.

### 4.3. Ethical Implications of Assault Prevention Technologies

While technology offers significant promise in enhancing personal safety, it also presents critical ethical challenges. Privacy concerns arise from the continuous tracking and data collection by IoT devices and mobile apps, risking breaches if not properly encrypted or anonymized [17,66]. Surveillance overreach may compromise autonomy, particularly in children and vulnerable populations, where GPS-based monitoring can suppress independence [21]. Moreover, algorithmic bias in AI models trained on unbalanced data may lead to false alarms or exclusion of marginalized users, demanding fairness in design [22,66]. The potential for misuse is another concern, as abusers may repurpose safety tools for control, surveillance, or intimidation, highlighting the need for tamper-resistant mechanisms and informed consent [19]. Studies also show that technologies often reflect a one-size-fits-all approach, neglecting intersectional factors, like race, class, or gender identity, which can deepen existing inequalities [18]. To ethically deploy such systems, designers must prioritize secure data handling, transparency, inclusivity, and user empowerment.

## 5. Conclusions

The SOS framework combines real-time AI applications, IoT, edge computing, and mobile technologies to establish an emergency detection and response system for health care and personal safety. By integrating wearable sensors, AI-based distress detection, and secure cloud communications, the framework guarantees fast, reliable, and highly scalable solutions for countless emergencies. Its wide array of applications in the field of hospitals, smart cities, public transportation, and law enforcement serves to showcase how the system’s versatility functions to enable safety and faster responses. Though there are challenges, like false-positive alerts, network constraints, and issues in user acceptance, advancements in AI accurateness, in hybrid cloud–edge processing along with awareness programs, could add more weight to the consideration of such systems. With strong encryption in place, GDPR/HIPAA compliance supported, and blockchain-based data security, the framework assures privacy and trust in the management of an emergency response. Overall, the SOS framework is a technologically advanced, effective, and life-saving solution for healthcare emergencies and assault prevention that will make communities safer and more responsive to critical situations.

## Figures and Tables

**Table 1:** Advantages and critical challenges of IoT devices in personal safety.

	Critical Analysis	Feature	Disadvantage
Personal Safety	The WE-Safe sensor network offers a low-power, solar-powered wearable solution with real-time monitoring and robust LoRa communication for hazardous environments. While innovative, it faces challenges, like reliance on sunlight and limited gateway functionality, with future potential in edge computing and expanded health applications [8].	The WE-Safe IoT project introduces a self-powered wearable sensor network using energy harvesting and low-power components, like the BME680 sensor, ATmega328p MCU, and LoRa communication. It ensures continuous environmental monitoring and real-time alerts via a mobile app, enhancing safety in hazardous conditions [8].	The WE-Safe sensor network, despite its innovation, faces key drawbacks, such as reliance on solar energy, which can limit reliability in low-light conditions, and basic gateway functionality that restricts dynamic control. Additional challenges include potential latency from LoRa communication, the need for broader real-world testing, complex power management, and dependence on user interaction for alerts [8].
Attack Prevention	An effective IoT cybersecurity approach by integrating hybrid optimization (GWO and Whale Optimization) with deep learning to enhance intrusion detection. It addresses key challenges, like malware classification and data dimensionality, showing improved accuracy and robustness—marking a significant advancement in securing IoT systems against evolving threats [31].	Feature selection is crucial for improving Intrusion Detection Systems (IDS) in IoT by identifying the most relevant data features, reducing noise, and enhancing detection accuracy. The paper highlights filter- and wrapper-based approaches, emphasizing bio-inspired methods, like Grey Wolf Optimization (GWO), to optimize feature selection and improve IDS performance in IoT security [31].	Its vulnerability to cyber-attacks due to insufficient intruder recognition systems and exposure to threats, like illegal downloads and viruses. It also highlights the complexity of feature selection in large-scale networks, posing challenges to optimizing intrusion detection systems [31].
Geographic Information System	The framework offers a comprehensive, technology-driven approach to crime prevention and women’s safety, emphasizing integrated crime analysis, societal participation, and data-driven insights. However, its implementation faces challenges, such as addressing crime hotspots and ensuring sustained community engagement, which need further refinement for real-world effectiveness [36].	The wearable device, integrated with the LoNET808 module, ensures reliable GPS/GSM connectivity and SOS alert functionality even without a smartphone. Paired with the “SpotHer” app, it enables real-time location sharing, volunteer navigation, and emergency support through a secure, community-driven network enhancing women’s safety through proactive societal involvement [36].	Mobile applications for women’s safety often lack reliability due to their dependence on user-reported data rather than verified crime records, and many fail to proactively warn about danger-prone areas. Their reactive nature, lack of integrated prevention strategies, and minimal societal involvement further limit their effectiveness in real-world safety scenarios [36].
Wearable Device for Safety	A robust IoT-based safety and health monitoring system using a dual-layer WBAN-LPWAN architecture, emphasizing data security through encryption and user authentication. It effectively monitors environmental and physiological parameters and includes an alert mechanism for emergencies, though scalability and user experience warrant further exploration [45].	The research paper proposes a hybrid wearable sensor network combining WBAN and LPWAN for outdoor safety and health monitoring. It uses BLE for on-body data collection and LoRa for long-range communication, enabling real-time access to physiological and environmental data via a REST web service [45].	The proposed IoT system faces challenges, like high power consumption, BLE’s limited range, integration complexity, and reliance on stable internet connectivity. Concerns also include data security, sensor accuracy under environmental interference, and high implementation costs, which may hinder widespread adoption [45].
Mobile Application for Safety	The paper critically reviews challenges in addressing violence against women and children (VAW/VAC), emphasizing AI fairness to prevent biased outcomes. It advocates for integrated research on VAW and VAC and highlights the role of technology in shaping effective prevention and intervention strategies [68]	The paper explores machine learning features for detecting violence against women and children, ranging from high-level visual cues to sensor data, like accelerometer and gyroscope signals. It highlights tailored feature engineering based on data types and underscores the role of computer science in advancing abuse detection technologies [68].	The effectiveness of safety devices often hinges on unpredictable bystander intervention and raises privacy concerns due to location and visual monitoring. Additionally, their lack of discretion and potential misuse for tracking can limit practicality and pose ethical risks [68].

**Table 2:** Performance, power consumption, and cost comparison of various personal safety technologies and systems.

Study	Technology/System	Performance	Power Consumption	Cost
Sathyasri et al. [7]	GPS, GSM, IoT safety device with neurostimulator	Real-time location tracking, emergency alerts	Moderate (GPS, GSM, shock)	Not specified
Wu et al. [8]	WE-Safe wearable sensor network	Good coverage (520 m), real-time alerts	Ultra-low, solar-powered	Moderate (energy harvesting tech)
Rodríguez et al. [9]	IoT + ML IPV management system	Ongoing surveillance, real-time risk analysis	Not specified	Not specified
Salami et al. [11]	SOS-FCI mutual authentication scheme	Improved security under attacks	Slightly increased under load	Not specified
Bhatia et al. [12]	MFEW anomaly detection system	99.8% accuracy (NSL-KDD dataset)	Not specified	Not specified
Zahra et al. [14]	GLSF2IoT with Fuzzy Logic	High accuracy in attack detection	Minimal overhead	Low
Mohsin et al. [17]	Smart Emergency Response System	99% emergency detection accuracy, 3 ms response time	Moderate (multiple sensors used)	Not specified
Márquez-Sánchez et al. [41]	AIoT multisensor bracelet and helmet	92% activity classification accuracy	Medium (real-time ML)	Moderate
Anandhi et al. [45]	IoT-based wearable safety device	Effective emergency signaling	Low	Low (lightweight and economical design)
Magidwar et al. [47]	Wearable safety jacket	Real-time alerts, audio/video capture	High (shock, GPS, GSM modules)	Moderate to high (includes multiple systems)
Islam et al. [55]	FSR-integrated wearable for harassment detection	99.3% accuracy	Low	Affordable (wearable prototype)
Revathi et al. [57]	Nerve Stimulator + ML threat detector	Multimodal safety detection	Moderate	Affordable
Karmakar et al. [63]	FemmeBand (EMG and HR sensors, WBAN)	Improved emergency alert efficiency	Low	Not specified
Yadav et al. [66]	WoSApp mobile app (shake-activated SOS)	Positive pilot results, GPS alerting	Very low	Low
Vinarao et al. [67]	GPS-SMS-based emergency mobile application	Reliable and efficient	Low	Low

## Data Availability

It is a review article and no new data were created.
